# Severe active C3 glomerulonephritis triggered by immune complexes and inactivated after eculizumab therapy

**DOI:** 10.1186/s13000-016-0547-6

**Published:** 2016-10-07

**Authors:** Tanja Kersnik Levart, Dušan Ferluga, Alenka Vizjak, Jerica Mraz, Nika Kojc

**Affiliations:** 1 Department of Nephrology, Division of Paediatrics, University Medical Centre, Bohoričeva 20, 1000 Ljubljana, Slovenia; 2 Institute of Pathology, Faculty of Medicine, University of Ljubljana, Ljubljana, Slovenia

**Keywords:** C3 glomerulonephritis, Eculizumab, Membranoproliferative glomerulonephritis, Complement alternative pathway dysregulation

## Abstract

**Background:**

Understanding the role of alternative complement pathway dysregulation in membranoproliferative glomerulonephritis (MPGN) has led to a dramatic shift in its classification into two subgroups: immune complex-mediated MPGN and complement-mediated MPGN, consisting of dense deposit disease and C3 glomerulonephritis (C3GN). A limited number of C3GN cases have been published to date with not yet conclusive results since the novel therapeutic approach with eculizumab was introduced.

**Case presentation:**

We report the clinical follow-up of a 16-year-old patient in whom a diagnosis of C3GN was confirmed by immunofluorescence and electron microscopy in second and third kidney biopsies, while the first biopsy revealed idiopathic immune complex-mediated MPGN type III, Anders and Strife variant, which failed to improve after several attempts at conventional immunosuppression therapy. Although applied late in an already fairly advanced stage of the severe active form of MPGN, the efficacy of eculizumab on C3GN was evidenced clinically and pathohistologically. Its beneficial influence on pathomorphogenesis was demonstrated by a unique follow-up in the last three biopsies, despite the recent observation, confirmed in this study, of eculizumab binding within the kidney tissue.

**Conclusions:**

Clinicians and pathologists should be aware that, in some patients, an underlying genetic or acquired complement alternative pathway abnormality can be masked by an initial immune complex-mediated mechanism, which subsequently triggers an unbalanced excessive continual driving of complement terminal pathway activation and the development of C3GN. In such a patient, supplementary steroids in addition to eculizumab appear necessary to achieve an adequate response.

## Background

Membranoproliferative glomerulonephritis (MPGN) shows a distinct histopathological pattern of glomerular injury but has many potential causes. Recent elucidation of the possible pathogenesis of MPGN had led to its new classification, into immune complex-mediated and complement-mediated diseases [1, 2]. The first is driven by classical complement pathway activation, while the second is believed to be associated with complement alternative pathway (AP) dysregulation and is a new entity, C3 glomerulopathy [3]. The latter embraces dense deposit disease (DDD) and examples of MPGN type I and III in which immunofluorescence reveals exclusive or predominant C3 deposits, now termed C3 glomerulonephritis (C3GN) [1–7].

Predominant C3 deposits detected by immunofluorescence define C3 glomerulopathy but its original definition as “C3 only” appeared too stringent if the goal of the diagnosis is to identify all candidates for evaluation of complement AP dysregulation. A new definition of C3 glomerulopathy was therefore proposed when C3 dominance is at least two orders of magnitude stronger than any other immune reactant [4, 8]. C3GN comprises in addition to MPGN also other histomorphologic patterns [4].

We describe a clinical case of a 16 year-old boy with C3GN, mediated by complement AP dysregulation, which appeared to be triggered by immune complex-mediated MPGN. He was successfully treated with eculizumab after conventional immunosuppression failed to result in clinical and laboratory improvement, but the immune complexes had disappeared in the second kidney biopsy, redefining the renal disease as C3GN.

## Case presentation

### Clinical history and initial laboratory data

A previously healthy boy presented at the age of 16 with edema, headache and paleness. He was hypertensive (RR 164/110 mmHg), with no other abnormalities on physical examination.

Nephrotic syndrome was confirmed (edema, proteinuria 4 - 18 g/day, hypoalbuminemia 21 – 28 g/L, typically changed proteinogram and lipidogram) with some additional elements of nephritic syndrome (microhematuria, hypertension). He was anemic but had normal platelets. LDH was normal at that time (4.1 mckat/L) but rose to twice as much very shortly afterwards. Serum creatinine was normal at disease presentation (73 μmol/L), as were liver tests and coagulation. He had ascites, enlarged and hyperechogenic kidneys and left ventricular hypertrophy. Follow-up values of laboratory parameters in relation to therapeutic approaches are given in Table 1.

**Table 1:**
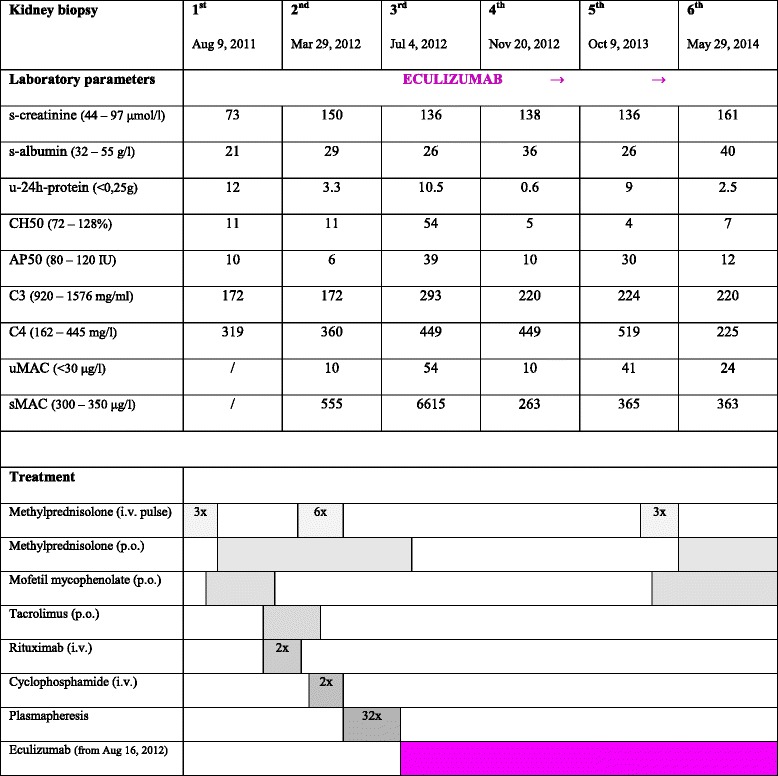
Laboratory parameters in relation to treatment modalities

*Legend for normal values:*

s-creatinine 44 – 97 mcmol/L; s-albumin 32 – 55 g/L; u-24 h-protein <0,25 g/day; CH50 – total hemolytic classical complement pathway 72 – 128 %; AP50 – total hemolytic alternative complement pathway 80 – 120 E; C3 – C3 complement factor 920 – 1576 mg/L; C4 – C4 complement factor 162 – 445 mg/L; uMAC – complement lytic complex in urine <30 mcg/L; sMAC – complement lytic complex in serum 300 – 350 mcg/L

*Legend to the treatment:*

Methylprednisolone 3x pulse 10 mg/kg/day i.v. (Aug 12, 13, 14, 2011)

Methylprednisolone 1 mg/kg/day p.o. (Aug 15 – Sep 22, 2011), thereafter slowly tapering dose

Mofetil mycophenolate 2 x 1000 mg/day p.o. (Sep 10 – Dec 22, 2011)

Resochine 1 tbl/day p.o. (Sep 20, 2011 – Sep 6, 2012)

Tacrolimus kept at a desired level 5 – 7 mcg/L p.o. (Nov 22, 2011 – Jan 5, 2012)

Rituximab 2 x 875 mg i.v. (Feb 3, 2012 and Feb 17, 2012)

Cyclophosphamide 1000 mg i.v. (Apr 2, 2012) + 750 mg i.v. (Apr 30, 2012)

Methylprednosolone 6x pulse 1 g i.v. (Apr 4, 5, 6, 7, 8, 9, 2012)

Methylprednisolone 0,5 mg/kg/day p.o. (Apr 10 – May 15, 2012), thereafter slowly tapering dose)

Plasmapheresis with fresh frozen plasma 32 sessions (May 3 – Aug 9, 2012)

Eculizumab 900 mg/week 3-times, thereafter 1200 mg/2 weeks; ongoing (Aug 16, 2012)

Methylprednosolone 3x pulse (Feb 24, 25, 26, 2014)

Methylprednisolone 1 mg/kg/day p.o. (Feb 27 – Apr 3, 2014), thereafter slowly tapering dose, kept at 8 mg/2^nd^ day

Mofetil mycophenolate 2 x 1000 mg/day p.o. (Apr 3, 2014 - ongoing)

### Kidney biopsies, diagnosis and clinical follow-up

**Fig. 1:**
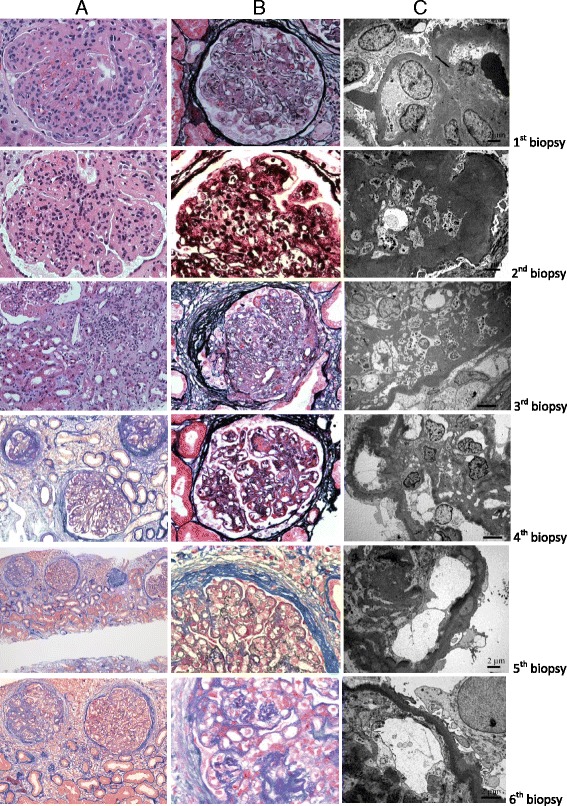
Light and electron microscopic images of 6 successive renal biopsies compared with various therapies. (1A-C) Initial biopsy with immune complex immunofluorescence pattern showing severe glomerular endocapillary proliferation and leukocyte exudation (**a** – H&E), glomerular basement membrane double contours (**b** – methenamine silver), prevailing transmembranous and scattered hump-shaped deposits (C – electron micrograph). (2A-C) On conventional immunosuppressive therapy in the second biopsy with highly dominant C3 immunofluorescence, severe glomerular proliferation, leukocyte exudation with pronounced lobularity (**a** – H&E), extensive capillary wall mesangial interposition with glomerular basement membrane double contours and disruption (**b** – methenamine silver), evidenced also on electron micrograph (**c**). (3A-C) On rituximab and plasmapheresis in the third biopsy, only slightly less active C3 membranoproliferative glomerulonephritis type III of Anders and Strife but significantly increased interstitial fibrosis with tubular fatty degeneration and cholesterol crystalline clefts, fibrocellular crescents and glomerulosclerosis (**a** – H&E, B – methenamine silver, **c** – electron micrograph). (4A-C) After initiation of eculizumab, while interstitial fibrosis, focal segmental glomerulosclerosis (**a** – AFOG trichrome) and mesangial-transmembranous deposits persist, a significant decrease in glomerular hypercellularity, active crescents and disappearance of leukocyte infiltration and necrotizing lesions are visible on methenamine silver stained section (**b**) and electron micrograph (**c**). (5A-B) With ongoing eculizumab therapy but withdrawal of conventional immunosuppression associated with the reappearance of immune complex immunofluorescence, similar histopathology as in the fourth biopsy (**a** – AFOG trichrome) but more pronounced refractile red stained glomerular capillary wall and mesangial deposits share some similarities to those of dense deposit disease (**b** – AFOG trichrome) and on the inner aspect of transmembranous deposits interrupting powdery dense deposits ascribed to eculizumab binding are visible on electron micrograph (**c**). (6A-C) After ongoing eculizumab and methyprednisolone therapy, chronic C3 glomerulonephritis presents similarly as in the fifth biopsy, with significant focal segmental glomerulosclerosis and interstitial fibrosis (**a** – AFOG trichrome), a lower level of glomerular proliferation and absence of active glomerular inflammation (**b** – AFOG trichrome) but with continuous powdery electron dense deposits (**c** – electron micrograph)

**Fig. 2:**
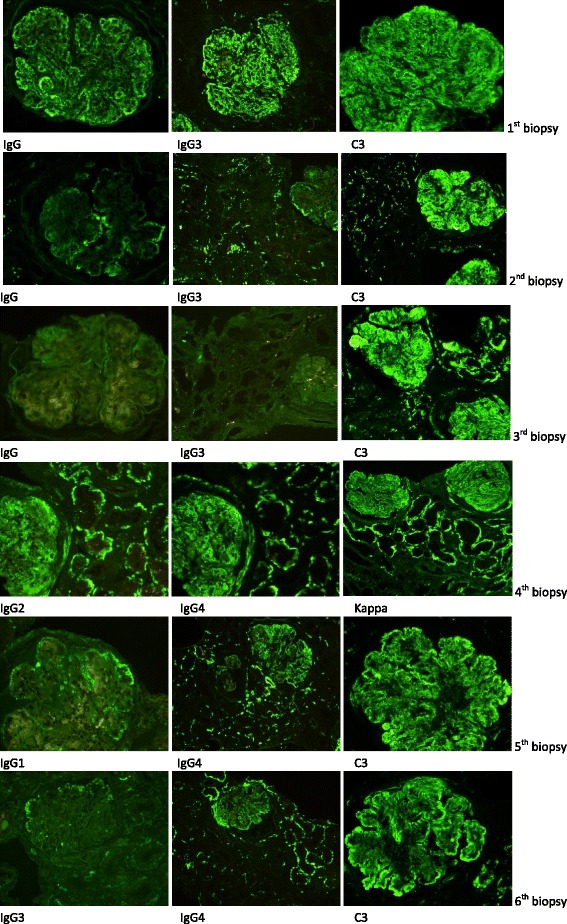
Immunofluorescence microscopic images of six successive renal biopsies compared with various therapies. (First biopsy) Granular glomerular mesangial and particularly capillary wall immunofluorescence moderate staining for IgG, subclass IgG3 and intense bright staining for C3. (Second biopsy) Scanty segmental glomerular granular staining for IgG, subclass IgG3, and intense staining for C3. (Third biopsy) Negative immunofluorescence for IgG, subclass IgG3, and pattern and intensity of C3 as in previous two biopsies. (Fourth biopsy) After introduction of eculizumab therapy immunofluorescence showing moderate granular mesangial and capillary wall as well as extraglomerular vessel and extensive tubular staining for IgG2, IgG4, kappa. (Fifth biopsy) On persisting eculizumab therapy but withdrawal of conventional immunosuppression reappearance of immune complex pattern expressed as segmental granular staining for IgG1, while moderate granular glomerular, extraglomerular vascular and tubular staining for IgG4 and intense bright staining for C3 persist. (Sixth biopsy) After ongoing eculizumab and methyprednisolone therapy immunofluorescence showing only segmental scanty granular staining for IgG3, persisting glomerular and extraglomerular staining for IgG4 and still intense staining for C3

The first kidney biopsy, ten days after initial clinical presentation, showed immune complex-mediated MPGN, classified as type III, Anders and Strife variant. Detailed findings of light microscopy, immunofluorescence and electron microscopy findings are given in Table 2, including five consecutive kidney biopsies, while representative pictures are presented in Figs. 1 and 2.

**Table 2:**
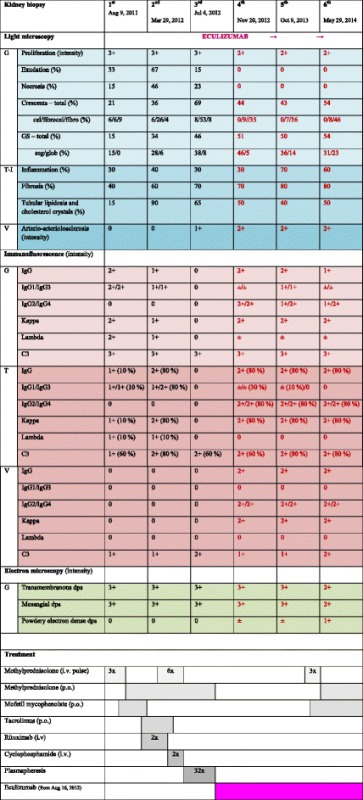
Light microscopy, immunofluorescence and electron microscopy results of six successive renal biopsies in relation to treatment modalities

*Legend:* % – proportion of glomeruli with lesion and estimated semi-quantitative tubulo-interstitial involvement, respectively; intensity – semi-quantitative values 0-3+; crescents active – cellular and fibrocellular; crescents inactive – fibrous; eculizumab treatment from August 16, 2012 ongoing

*Abbreviations: G* glomerular, *T-I* tubulo-interstitial, *V* vascular, *cel* cellular, *fibrocel* fibrocellular, *fibro* fibrous, *GS* glomerulosclerosis, *seg* segmental, *glob* global, *dps* deposits

We found low activity of alternative and classical complement pathways, low C3 and normal C4, CFH, CFI and CFB. C3 nephritic factor (5 %; ref.: <10 %) and anti-CFH antibodies (46 AU/ml; ref.: <110 AU/ml) were negative. Anti-C1q antibodies (93 IU/ml; ref. <15 IU/ml) were positive, while all other laboratory examinations for possible autoimmune disease were negative. Cryoglobulinemia, paraproteinemia and chronic infections were ruled out.

The boy was initially treated for suspected lupus nephritis, although he never fulfilled ARA criteria for systemic lupus erythematosus. The detailed treatment is described in Table 1. Despite all conventional immunosuppressive therapeutic approaches, the boy remained heavily nephrotic, with persistent malignant hypertension (RR 180 – 190/100 – 110 mmHg) on 7 antihypertensive drugs, severe dyslipidemia and slowly increasing s-creatinine. One month after the last dose of rituximab, with no clinical and laboratory improvement, a second kidney biopsy was performed. It showed decreased IgG but persisting bright C3 staining, thus fulfilling the diagnostic criteria for C3GN and changing the pathohistological diagnosis from immune complex-mediated to complement-mediated MPGN III (Table 2, Figs. 1 and 2). This was even more evident in the third biopsy, in which IgG deposition had disappeared entirely, while C3 remained unchanged. Before starting eculizumab, one year after the first clinical symptoms, severe glomerular inflammatory activity with nearly 70 % of mostly active cellular and fibrocellular crescents was already associated with 46 % of mostly segmental glomerulosclerosis and 70 % of interstitial fibrosis (Table 2, Figs. 1 and 2). The patient was still heavily nephrotic. Low serum C3 values associated with extremely high (6615 mcg/L) serum membrane attack complex (sMAC) suggested that dysregulation of complement AP was responsible for the ongoing nephrotic syndrome. The patient was therefore put on eculizumab after the third biopsy. In addition to completely normalized sMAC (263 mcg/L) after two months of treatment, associated continuing improvement of renal function and proteinuria was evident (Table 1). After initiation of eculizumab, the fourth renal biopsy revealed a significant decrease of glomerular inflammatory activity (complete disappearance of glomerular infiltration by neutrophils and necrotizing lesions, significantly reduced glomerular proliferation rate), the latter being sustained throughout the treatment, as demonstrated by the fifth and six renal biopsies (Tables 1, 2, Figs. 1 and 2). Furthermore, newly developed cellular crescents were no longer found and a gradual decrease or switching of fibrocellular to completely inactive fibrous crescents, associated with partial replacement of segmental with global glomerulosclerosis, was observed in follow-up biopsies after eculizumab was introduced.

In the fourth biopsy, we noticed de novo staining for IgG2, IgG4, kappa and traces of IgG1, IgG3 reappeared in association with gradually worsening proteinuria, while the patient was off all conventional immunosuppression drugs. A fifth biopsy was performed, showing intensified IgG1, IgG3 staining and persisting staining for monoclonal IgG2, IgG4, kappa (Table 2, Fig. 2). Reintroducing conventional immunosuppression with steroids, in addition to eculizumab, resulted in clinical and laboratory improvement, which is still being sustained. The final kidney biopsy showed persistently suppressed glomerular inflammatory activity, slightly increased glomerulosclerosis and a decrease of IgG1, IgG3 deposits, while monoclonal IgG2, IgG4, kappa remained virtually unchanged and C3 deposits remained of the same intensity in all 6 biopsies, even when eculizumab was introduced (Table 2, Fig. 2). While extensive interstitial fibrosis, already present before introducing eculizumab, persisted at a similar rate, a gradually increasing intensity of interstitial inflammation was observed, accompanied by tubular lipidosis and cholesterol crystalline deposits, as well as partial switching of segmental to global glomerulosclerosis (Table 2, Fig. 1).

Molecular genetic analysis showed no evidence of disease-causing mutations in the genes coding for C3, CD46, CFB, CFH, CFHR1, CFHR2, CFHR3, CFHR4, CFHR5, CFI and THBD but the patient was found to be heterozygous for four *CFH* single nucleotide polymorphisms. These polymorphisms included exon 9 c.1204C > T p.H402Y (CAT > TAT), as well as exon 7 c.921A > C p.A307A (GCA > GCC), exon 13 c.2016A > G p.Q672Q (CAA > CAG) and exon 18 c.2808G > T p.E936D (GAG > GAT).

## Discussion

A clinical case of a 16-year-old boy with presumed combined immune complex-mediated and complement-mediated MPGN is described. At the first renal biopsy, immune complex-mediated MPGN, classified as type III, variant Anders and Strife, was found associated with dominant 3+ staining for C3 and 2+ staining for IgG, subclasses IgG1 and IgG3. According to the definition of C3GN, characterized by C3 dominance of at least two orders of magnitude of intensity more than any other immune reactant [4, 8], these findings did not fulfill the criteria for C3GN. After multiple conventional immunosuppression approaches applied to the patient, while bright C3 staining persisted, IgG diminished and finally disappeared, which gave a diagnosis of C3GN in the second and following biopsies. It has already been proposed that, in some patients, MPGN is initiated by immune complexes but the disease is accelerated by complement AP dysregulation [5, 9]. It can be hypothesized that activation of the complement classical pathway, initiated by immune complex deposition, might through the release of C3b, a constituent of AP C3bBb convertase, have augmented and perpetuated the activation of dysregulated complement AP. Although it has been described that patients with C3GN might suffer from mutation in one or more of the complement genes or produce auto-antibodies against the complement regulatory proteins and/or AP C3 convertase [2–4, 10, 11], we could not find any disease-causing mutation, nor auto-antibodies associated with complement AP dysregulation in our patient. We did however find polymorphism in the *CFH* gene, which has already been described in some patients with DDD [12], as well as *CFH* polymorphisms evidenced in patients with atypical hemolytic uremic syndrome [13, 14].

There are 11 reported cases on the use of eculizumab for treatment of C3GN, mostly but not always with good results [15–25]: 7 cases in native kidneys and 4 as a recurrence in transplant kidneys. Of the 7 cases, one was immune complex MPGN type I, refractory to conventional immunosuppression, with complement analysis strongly suggesting AP activation, positive C3 nephritic factor and several polymorphisms in complement regulators on genetic testing but without re-biopsy after eculizumab [15]. Furthermore, Kerns et al. [20] described a similar patient to ours in whom the initial kidney biopsy revealed immune complex-mediated MPGN, although without detailed subtyping, and in whom standard immunosuppression resulted in histologic transformation into C3GN, but without biopsy follow-up after eculizumab treatment. Our patient had a much longer clinical and histological follow-up, with six successive kidney biopsies, the last three after eculizumab had been introduced. It has been suggested that MPGN III of Anders and Strife, identified in our case, often represents C3GN [4]. In addition, it must be noted that complement AP abnormalities, genetic or acquired, have been identified in a subset of patients showing susceptibility to systemic lupus erythematosus [5], and particularly frequently in patients with immune complex-mediated MPGN [26] and atypical post-infectious glomerulonephritis [27], the latter showing overlapping features with C3GN [28].

Since eculizumab is a humanized monoclonal antibody that binds with high affinity to C5 and prevents the generation of MAC and release of the very potent inflammatory mediator C5a, it may provide effective targeted treatment for patients with C3GN sharing in common an abnormality in the regulation of complement AP [29]. However, it has been suggested that eculizumab might be effective in some but not all cases of C3GN, and that elevation of sMAC, short disease duration, acute lesions and limited fibrosis before treatment may predict a favorable response [2, 4, 22]. The benefit of eculizumab was confirmed in our patient not only with complete normalization of previously highly increased sMAC but also with improvement of the clinical features and laboratory findings. Furthermore, a significant lowering of glomerular inflammatory activity was demonstrated in the last three follow-up biopsies by the disappearance of neutrophil infiltration and necrotizing lesions, switching of active to inactive fibrous crescents and a decrease in glomerular proliferation intensity. Glomerulosclerosis only slightly increased, associated with a gradual partial replacement of a segmental with a global type of this scarring lesion, probably related to some extent also to non-immune factors, particularly arterial hypertension. Extensive interstitial fibrosis had mostly been established before eculizumab was introduced. Tubular fatty degeneration and cholesterol crystalline clefts can be ascribed to severe hyperlipidemia. Furthermore, interstitial inflammation is presumably also unrelated to basic complement AP dysregulation but caused by other secondary mechanisms in chronic glomerular diseases.

As in the presented case, the literature shows that prescription of eculizumab in C3GN is frequently delayed until alternative diagnostic possibilities have been ruled out and conventional immunosuppressive approaches have failed [30]. The question arises of when it is too late to initiate such expensive and potentially long-term treatment. Clinical and biopsy follow-up in our patient convincingly demonstrated that, although delayed, institution of eculizumab therapy to a patient with already fairly advanced active C3GN, with nearly 70 % of mostly active crescents, resulted not only in clinical improvement but also in a persistent significant decrease in glomerular inflammatory activity and cessation of further progression of renal disease, as shown in the last three follow-up biopsies. Our observation is in line with reports of recovery of renal function and decrease of proteinuria rate in one patient with crescentic, clinically rapidly progressive GN [24] and in another similar patient already on dialysis, which could be discontinued after 5 months of eculizumab therapy [25]. It appears that, in such cases, a minimum of six months of eculizumab application might be necessary prior to declaring treatment failure [25, 30]. However, it has to be considered that case reports might be biased by selective reporting of positive results. Non-selective case reports of the rare disease and, especially, randomized prospective controlled studies are expected to provide accurate results about the efficacy of eculizumab in C3GN.

It has already been reported that in biopsies performed after eculizumab treatment, there was de novo staining for monoclonal IgG2, IgG4, kappa with the same distribution as C3 and C5b-9, suggesting the binding of the humanized monoclonal antibody eculizumab onto preexisting complement deposits in the renal tissue [18]. At the same time, there was a marked decrease in inflammatory activity observed post-treatment although staining for C3 and C5b-9 appeared largely unchanged in the pre-treatment and post-treatment biopsies [18]. Similar results were obtained in our patient in post-treatment biopsies, with de novo staining for monoclonal IgG2, IgG4, kappa and persistent unchanged staining for C3. Furthermore, the results of Herlitz et al. [18] after one year of therapy were confirmed in our study in all three of the last biopsies during a two-year follow up, without convincing evidence of any harmful influence of eculizumab tissue binding. However, the long-term clinical significance of this drug-tissue interaction still remains unclear. Long-term eculizumab use has been studied in patients with paroxysmal nocturnal hemoglobinuria and there was no evidence of the development of proteinuria or renal insufficiency [31]. The transitory worsening of the clinical picture and reappearance of IgG1 and IgG3 in kidney biopsy samples after several months of initially successful treatment with eculizumab in our patient can be explained by recurrence of the immune complex-mediated mechanism following the complete withdrawal of conventional immunosuppression. This is supported by the patient’s clinical and histomorphological improvement after methylprednisolone was reintroduced.

## Conclusions

We describe a patient with C3GN due to complement AP dysregulation, triggered by immune complex-mediated MPGN III, variant Anders and Strife, who was successfully treated with eculizumab after treatment failure with various conventional immunosuppression approaches. His clinical condition, however, deteriorated temporarily after several months of eculizumab treatment, probably due to the unsuppressed immune complex-mediated mechanism of his disease, identified by the reappearance of IgG deposits in biopsies when the patient was temporarily completely off all conventional immunosuppression drugs. It must be stressed that in primary immune complex-mediated glomerulonephritis, especially MPGN, which does not respond to immunosuppression, the presence of complement AP dysregulation should be considered. In such a case, special laboratory and molecular genetic tests must be done to explore the exact pathophysiology of the disease and target the treatment accordingly. Furthermore, our significantly longer biopsy follow-up study confirms the recent new observation of eculizumab binding to renal tissue without apparent evidence of its injurious consequences, although eculizumab deposits share some similarities with those of monoclonal immunoglobulin deposit diseases.

## Abbreviations

AP: Alternative pathway
ARA: American Rheumatology Association
C3: Complement factor 3
C3GN: C3 glomerulonephritis
C4: Complement factor 4
CD46: CD46 complement regulatory protein – cluster of differentiation
CFB: Complement factor B
CFH: Complement factor H
CFHR1-5: Complement factor H related proteins 1 - 5
CFI: Complement factor I
DDD: Dense deposit disease
LDH: Lactate dehydrogenase
MPGN: Membranoproliferative glomerulonephritis
sMAC: Serum membrane attack complex
THBD: Thrombomodulin
